# Toward developing a compact total artificial heart using a soft robotic fluidic transmission system

**DOI:** 10.1126/sciadv.adv4854

**Published:** 2025-07-02

**Authors:** Maziar Arfaee, Lucas C. van Laake, Shibo Zou, Charlotte Bording, Jolanda Kluin, Johannes T. B. Overvelde

**Affiliations:** ^1^ Cardiothoracic Surgery, Amsterdam UMC location University of Amsterdam, Amsterdam, Netherlands.; ^2^ Department of Cardiothoracic Surgery, Thorax Center, Erasmus MC, Rotterdam, Netherlands.; ^3^ Autonomous Matter Department, AMOLF, Amsterdam, Netherlands.; ^4^ Mechanical Engineering Department, Eindhoven University of Technology, Eindhoven, Netherlands.; ^5^ Department of BioMechanical Engineering, Delft University of Technology, Delft, Netherlands.; ^6^ School of Mechanical and Aerospace Engineering, Queen’s University Belfast, Belfast, UK.; ^7^ ICMS, Eindhoven University of Technology, Eindhoven, Netherlands.

## Abstract

Cardiovascular diseases are a leading cause of mortality, with limited possibilities for transplantation due to a critical shortage of donor hearts. Replacing the heart with total artificial hearts (TAHs) remains challenging, due to size constraints and energy requirements, among others. To address this, we introduce the LIMO heart, a compact TAH concept based on an efficient soft fluidic transmission system. By reducing actuator volume and enhancing energy transfer, LIMO enables a more compact and efficient design. We developed a soft ventricle prototype using thin-walled pouch actuators that achieve transmission ratios above one via circumferential shrinkage. A fast, cost-effective prototyping method accelerated testing. Experimental results showed high energy transfer efficiency (82 to 91%), and in vitro tests demonstrated promising cardiac outputs of 5.9 liters per minute against aortic pressure and 7.6 liters per minute against pulmonary pressure. These findings represent a step toward a more broadly applicable biventricular soft robotic TAH for treating end-stage heart failure.

## Teaser

The LIMO ventricle is a concept for a compact soft robotic TAH that leverages an efficient soft fluidic transmission system.

## INTRODUCTION

Cardiovascular diseases are a major cause of death globally, and as the population ages, the prevalence of heart failure is expected to increase (*1*, *2*). Heart transplantation remains the ideal treatment for severe heart conditions, but a critical shortage of donor hearts necessitates the development of alternative solutions such as left ventricular assist devices (LVADs) and total artificial hearts (TAHs). While LVADs provide notable support, they are not suitable for patients with severe biventricular failure and carry risks of complications such as post-LVAD right ventricular failure, highlighting the need for TAH development (*3*).

Currently available TAHs that are approved as a bridge to transplant are the SynCardia and Carmat, both fluid-driven (either pneumatic or hydraulic) pulsatile devices (*4*, *5*). The popularity of fluid-driven systems in developing TAHs and the need for more biomimetic behavior have opened the door for soft robotics as an alternative solution to traditional driving systems (*6*). Soft robotics is being used to develop different types of medical devices (*7*), such as wearable devices (*8*–*10*) and implantable devices (*11*, *12*), among which cardiovascular devices have gained significant attention over the past few years (*13*–*15*). Recent studies in the development of heart assistive devices (*16*–*20*) and TAHs (*14*, *15*, *21*) using soft robotics highlight a potential avenue to more closely mimic the natural behavior of the heart. In a recent study on developing a soft TAH, sufficient cardiac output (CO) of 8 liters/min has been achieved in a prototype working for 30 hours (*14*).

Although the results of recent studies on developing a soft TAH warrant further investigation, an important problem of all the currently existing fluidically activated TAH designs—including the soft TAH designs—remains their total size. The combination of the actuator, driving system, and power supply leads to bulky devices that cannot be fully implanted, therefore requiring percutaneous drivelines (*2*). Existing devices require at least the same amount of volumetric input to the actuators compared to the volumetric (blood) output ejected. This means that the actuators need to displace a large amount of fluid (or air) that is identical to or larger than the volume of blood that needs to be pumped. Besides that, a reservoir is needed to store the actuation fluid that is not inside the actuators of the device. In comparison, in a natural heart, the cardiac tissue only takes up approximately equal or slightly larger space compared to the total volume of blood that needs to be pumped in each cycle (*22*–*25*). In addition, the membrane-based actuators of these soft TAHs work only effectively at relatively high pressures (150 to 200 kPa) (*14*), which are more than 10 times higher than the required blood pressure. This considerably limits the energy efficiency of soft TAHs and increases the size of auxiliary components such as batteries and pumps.

As a result, the existing driving system for (soft) TAHs still requires a bulky driving system to work. This same problem limits both the miniaturization of the SynCardia and Carmat, because they classify as membrane-based systems that can reach a 1:1 fluidic transmission ratio (volumetric input/output) at best. Carmat only partially circumvents this problem by alternatingly activating the left and right ventricles, while SynCardia’s drive system can only be placed extracorporeally. In mechanical or electrical systems, this problem would normally be overcome using a transmission system, such as mechanical gears or electrical transformers. Therefore, a natural question to ask is what different types of efficient soft fluidic actuation strategies can be used that achieve fluidic transmission, with the ultimate goal to reduce the size of the complete TAH system and increase the application potential for TAHs in general.

We propose a soft ventricle that can be used in a fully soft TAH concept that we refer to as the LIMO (Less In, More Out) heart. The actuation of the ventricle is based on existing pouch motors (*26*–*28*). Our concept consists of a blood-collecting chamber (a so-called ventricle) that is surrounded by flat pouches that transform into cylinders when actuated. The aim of our soft ventricle design is to achieve fluidic transmission with only limited energy loss (i.e., high efficiency). In essence, we aim to build a system where lower volumes of fluid (gas or liquid) are delivered at higher pressures as input to the actuators, which are then transformed into a larger blood output volume at the required reduced pressures (movie S1). We anticipate that such an efficient pressure-volume transmission strategy reduces the total size of a TAH, as both the total fluidic volume [driving fluid and maximum stroke volume (SV) combined] and required pump power and battery size can be considerably reduced.

The remainder of this paper is divided into the following sections. First, we introduce our concept and an analytical model that theoretically explores the possibility of using only the geometry of the device design to achieve programmable fluidic transmission ratios that are higher than one. Second, we investigate experimentally the effect of geometry on the input pressure and force output of pouch arrays and how the results can be translated to the application in a soft TAH concept. Third, we describe the fabrication method of a closed chamber based on pouch motors and experimentally prove its functioning as a pump in a static experimental setup with afterload. We finalize by reporting the results of testing a single ventricle in a single-sided mock circulation loop (MCL) against physiological conditions, to explore the effect of dynamics and cyclic loading on the system.

## RESULTS

### Rationale and design of the LIMO heart

**Fig. 1.**
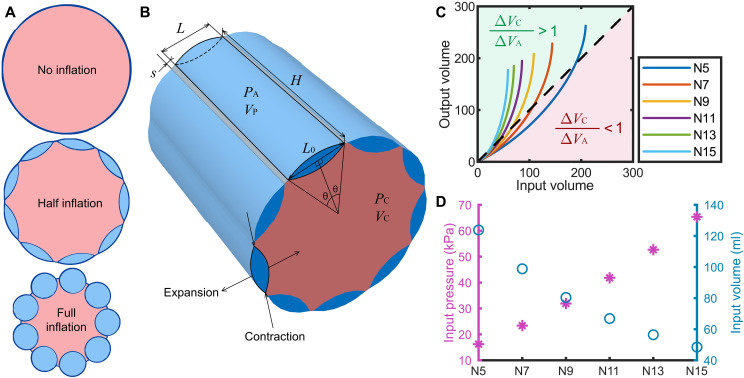
Simplified working mechanism of the proposed fluidic transmission system. (**A**) Cross-sectional view of a cylindrical chamber surrounded by vertical pouches and the inner deformation after pouch inflation. (**B**) Model of a single pouch with cylindrical surfaces. *P*_A_, actuator pressure; *V*_P_, volume of a single pouch; *P*_C_, ventricular pressure; *V*_C_, volume of the cylindrical ventricle. (**C**) Output-input geometrical volume relation obtained from analytical modeling. The fluidic transmission ratio (Δ*V*_C_/Δ*V*_A_) is higher than the one above the dashed line. Δ*V*_C_, output volume; Δ*V*_A_, input volume. (**D**) Input volume and pressure needed for samples with different numbers of channels to reach an output volume of 90 ml at 15 kPa, considering a cylindrical system with an initial diameter of 60 mm, a height of *H* = 100 mm, and a seam width of *s* = 1.5 mm.

We propose a model that consists of a cylindrical ventricle surrounded by pneumatically actuated pouches that are aligned vertically. By pressurizing the pouches, they deform from a flat shape into a cylindrical shape, which causes their (arc-)length to decrease (Fig. 1A). Therefore, the circumference of the cylindrical ventricle shrinks when the pouches are inflated while maintaining effectively the same vertical height (*29*). This global deformation accounts for the major proportion of the total volumetric shrinkage of the ventricle. In addition, approximately half of each pouch volume occupies the inner space of the ventricle, which also causes a reduction of the inner volume (Fig. 1A).

On the basis of these two observations, we can derive a simplified analytical model to qualitatively investigate the behavior of the system and to investigate the feasibility of attaining desired fluidic transmission ratios that are larger than one. The model assumes an idealized cylindrical initial shape of the ventricle, parametrized by height *H*, and a number of pouches *N* of initial length *L*_0_ that are separated by seams of width *s* (Fig. 1B). We furthermore assume that the height *H* remains constant, and the material is inextensible, such that the deformed shape is fully described by the opening angle θ of the pouches (*26*). The deformed length *L* and volume *V*_P_ of a single pouch can then be expressed as

$$\begin{array}{c} L=L_{0}\text{sinθ}/\theta \\ V_{P}=L_{0}^{2}H\left(\theta -\text{cosθ sinθ}\right)/\left(2\theta^{2}\right) \end{array}$$ (1)

We define *V*_A_ *= N V*_P_ as the total actuator volume, i.e., the volume of all pouches combined. On the basis of Eq. 1, we can approximate the radius *r*_C_ and volume *V*_C_ of the cylindrical ventricle as

$$\begin{array}{c} r_{c}=N\left(L+s\right)/\left(2\pi \right)\\ V_{c}=H\pi {r_{c}}^{2}-V_{A}/2 \end{array}$$ (2)

We can furthermore define the ejection fraction (EF), which is a common performance measure in the natural heart and in artificial heart devices

$$\text{EF}=\Delta V_{C}/V_{0}\times 100\%=\left(1-V_{C}/V_{0}\right)\times 100\%$$

where *V*_0_ is the initial volume of the cylindrical ventricle before actuation.

Excitingly, from this simplified geometrical model interpreted in Fig. 1C, we learn that the cumulative volume expelled from the ventricle Δ*V*_C_ can exceed the cumulative volume introduced into the pouches Δ*V*_A_. We can choose a desired fluidic transmission ratio

$$i=\Delta V_{C}/\Delta V_{A}$$

by selecting the number of pouches *N* (Fig. 1C). The fluidic transmission ratio *i* at full actuation (i.e., θ = π/2) can theoretically be chosen arbitrarily large by increasing *N*. This is because, as *N* increases, Δ*V*_A_ approaches zero, while Δ*V*_C_ approaches *V*_0_ (1 − 4/π^2^) ≈ 0.59 *V*_0_, resulting in EF > 59% for all values of *N*. Note that this relation is only valid if we assume that seam width *s* is negligible. We show the modeled effect of *N* and *s* on *i* and EF in fig. S7.

Next, we derive the required actuator pressure *P*_A_ that balances a given ventricular pressure *P*_C_ for different values of pouch opening angle θ. We assume static equilibrium and find the pressure ratio P* = *P*_A_/*P*_C_ by conservation of energy

$$\begin{array}{c} P_{A}{dV}_{A}+P_{c}{dV}_{c}=0\\ P^{*}=-{dV}_{c}/{dV}_{A}=-\left({dV}_{c}/\mathrm{d\theta}\right)/\left({dV}_{A}/\mathrm{d\theta}\right)\\ P^{*}=\left[N/\left(2\pi \text{cosθ}\right)\right]\left(\text{sinθ}+\theta s/L_{0}\right)+1/2 \end{array}$$ (3)

where we used Eqs. 1 and 2 for *V*_A_ and *V*_C_.

Using Eqs. 1 to 3, we determine the actuator input volume and pressure required to expel a representative physiological SV and afterload delivered by a healthy left ventricle, equal to a volume Δ*V*_C_ = 90 ml from the ventricle, against a constant afterload of *P*_C_ = 112.5 mmHg (15 kPa), which are in the range of average values for a heart working under normal conditions (*30*, *31*). For these fixed output requirements of the cylindrical ventricle, varying the number of pouches reveals an expected trade-off between actuator pressure and volume, where an increasing number of pouches require less input volume but higher actuator pressure (Fig. 1D).

Taken together, our model informs a rational selection of *N* for given application requirements, based on analytical relations between EF, required actuation volume *V*_A_, and required actuation pressure *P*_A_. Practically, there may be additional considerations for choosing *N*, such as fabrication limitations or a minimum channel width that is required to avoid excessive viscous losses when a driving fluid is forced in and out of the actuators.

### The effect of channel size on the response of pouch arrays

**Fig. 2.**
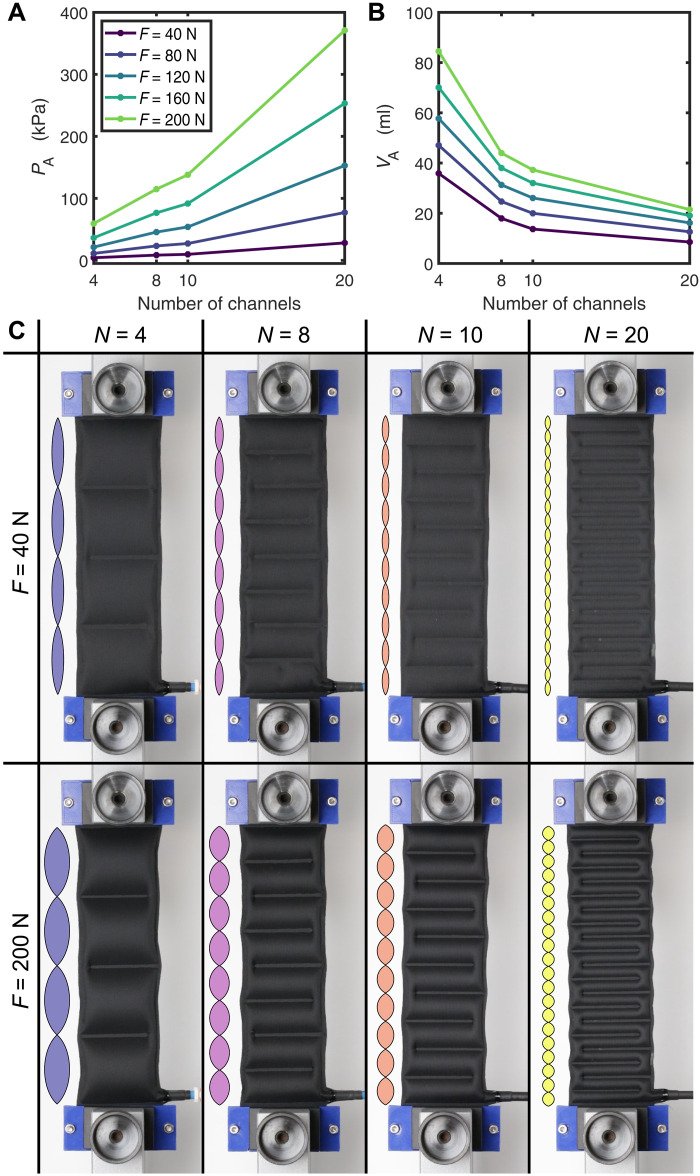
Blocked displacement testing of pouch arrays. (**A**) Input pressure (*P*_A_) needed to reach certain forces (*F*) for samples with different numbers of pouches (i.e., different pouch sizes). (**B**) Input geometric volume (*V*_A_) needed to reach certain forces for samples with different numbers of pouches. (**C**) From left to right, pouch arrays with 4, 8, 10, and 20 pouches. Top and bottom rows show the samples exerting 40 and 200 N to the jaws of the tensile testing machine. Pouch arrays with more (smaller) pouches require lower input volume to reach a certain force, at the cost of higher input pressure compared to pouch arrays with fewer (larger) pouches.

To explore the feasibility of implementing the fluidic transmission ratio experimentally, we first focus on fabricating and measuring the performance of pouch arrays. We are specifically interested in the relationship between the pouch actuator pressure and resulting circumferential forces that can be delivered by the pouches. To determine whether the number of pouches has an effect on the maximum force that can be delivered, we can layout the design of the proposed ventricles that are made from heat sealing two layers of nylon fabric coated with thermoplastic polyurethane (TPU-coated nylon) (Riverseal 70 LW, 78Dtex; 170 g/m^2^; Rivertex, Culemborg, The Netherlands) with varying numbers of pouches *N* to obtain flat arrays of pouch motors (Fig. 2) (*26*). We designed and performed so-called blocked displacement experiments (*27*), in which we clamp the samples in a tensile testing machine (INSTRON 5965, Norwood, United States) using custom-designed clamps, which hold the pouch motor’s ends at a fixed distance, while we quasi-statically increase the pressure (*P*_A_) in the pouch actuators (movie S2).

The measured results in Fig. 2A demonstrate that the number of pouches does not directly affect the ability to reach certain force levels. However, and as expected, as the surface area of the pouches is reduced for increasing *N*, we do require higher pressures in pouch arrays with more pouches to achieve these forces (Fig. 2A). The required higher pressures come with the benefit that less volume (*V*_A_) is needed to inflate the pouch motors for increasing *N* (Fig. 2B). Therefore, this experiment demonstrates the transmission trade-off between the pressure and volume needed to reach a certain force in samples with different geometries. Thus, we can obtain different behaviors by just changing the design while using the exact same amount of material (Fig. 2C).

### Fast and cost-effective prototyping method

While so far we have focused on a relatively simple geometry, fabricating a leak-proof soft artificial ventricle will lead to additional complexities that cannot easily be modeled. Given these complexities that are involved with soft and flexible components, it is beneficial to have a fast and cost-effective prototyping method to fabricate different designs of our pouch-based ventricle and investigate them experimentally. Therefore, we implemented a prototyping method that enables us to effectively fabricate several completely soft samples with different designs using only a customized three-dimensional (3D) printer computer numerical control (CNC) machine that can be used for both heat sealing (*26*) and 3D printing of TPU components.

**Fig. 3.**
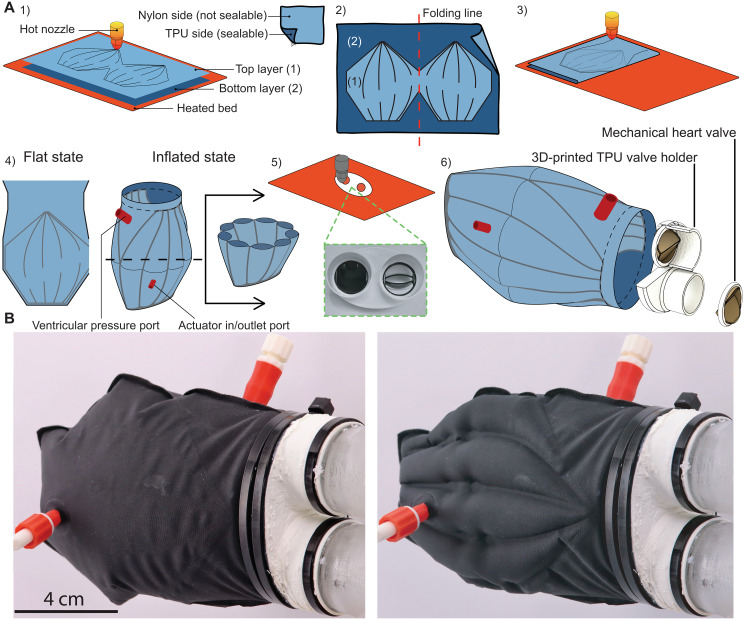
Fast and cost-effective fabrication method for the LIMO heart ventricle. (**A**) 1) Heat sealing two layers of TPU-coated nylon to create pouches. 2) Cutting leftovers from one side and folding the sample from its middle line. 3) Second heat sealing step to create a ventricle chamber. 4) Schematic 3D shape of the ventricle chamber after inflation. 5) 3D printing a TPU soft housing that holds the inlet and outlet valves and also acts as an interface between the prototype and the MCL setup. 6) Exploded view of all components. (**B**) LIMO ventricle in a relaxed and actuated state.

With this approach, we fabricate an artificial ventricle of our LIMO heart consisting of three major fabrication steps (Fig. 3): (i) heat sealing two layers of fabric together to create the pouches, (ii) cutting the outline of the artificial heart and revealing a layer of heat-sealable fabric to perform a second round of heat sealing to create the internal ventricular chamber, and (iii) 3D printing soft TPU connectors and a soft TPU housing for mechanical heart valve prostheses (Sorin Bicarbon, Sorin Group, Milan, Italy), which are sealed or glued to the ventricle directly (Fig. 3A and movie S3). Note that to create the ventricle (Fig. 3B), we use a 2D fabrication method (heat sealing) that results in an elliptic cross-sectional area (movie S4), rather than a perfect cylinder considered in our analytical model (Fig. 1B). The relaxed and actuated states of the ventricle are shown in Fig. 3B.

It should be noted that although this technique is cost and time efficient, it can now only be used in the proof-of-concept phase to evaluate the working mechanism. Using the same platform, we were also able to print soft valves that could directly be implemented in the ventricle to obtain a fully soft device (fig. S9). However, the material and heat sealing technique used for prototyping are not yet adequate for producing durable samples capable of withstanding the extreme cyclic loading required for real-world TAH applications. Notably, all observed failures occurred near the sealing lines, particularly at the end points adjacent to the gaps functioning as air channels between pouches (fig. S10). In our case, we were able to obtain in the order of 1500 to 2000 cycles per sample fabricated. This was sufficient to prove the concept and perform all the required measurements. Further optimization of the design to limit stress concentrations at sealing lines is essential to further develop the demonstrated fluidic transmission concept toward a TAH concept.

### In vitro quasi-static characterization of the artificial ventricle

**Fig. 4.**
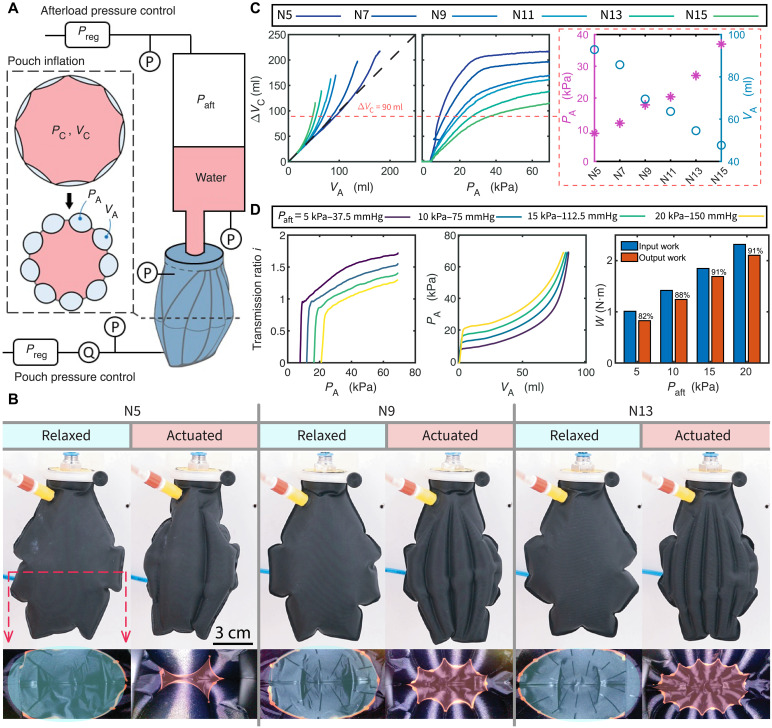
Quasi-static experiments to evaluate transmission ratio and efficiency. (**A**) Experimental setup, including a water tank, pressure regulators (*P*_reg_), pressure sensors (*P*), and an air flow sensor (*Q*). (**B**) Relaxed and actuated state of prototypes N5, N9, and N13, showing the difference in the internal deformation at maximum actuator pressure of *P*_A_ = 40 kPa against identical internal pressure of *P*_C_ = 10 kPa; light blue, inner surface area at the relaxed state; light red, inner surface area at the actuated state. (**C**) Experimental results of quasi-static testing of samples with different numbers of pouches against minimum afterload (3.8 kPa). Volumetric output (*∆V*_C_) at various actuator volumes (*V*_A_) and pressures (*P*_A_), showing the trade-off between *V*_A_ and *P*_A_ required to reach a certain volumetric output of 90 ml in different samples. (**D**) Experimental results of quasi-static testing of prototype N9 against various afterloads (*P*_aft_) between 5 and 20 kPa (37.5 to 150 mmHg).

We next aim to determine whether our fabricated prototype for a soft artificial ventricle can achieve higher fluidic transmission ratios than one, as predicted by our analytical model (Fig. 1C). We first conducted an experiment with a minimum afterload of 3.8 kPa (originating from the column of water) that we could achieve in our quasi-static experimental setup (Fig. 4A). We tested a range of artificial ventricles that are identical in size (*V*_0_ = 300 ml at the relaxed state against a minimum afterload) and only varied the number of pouches *N* = 5, 7, 9, 11, 13, and 15 by varying the length *L*_0_ of the individual pouches while keeping their height identical (Fig. 4B, fig. S3, and movie S4). Because the pouches occupy negligible space, the total volume of a biventricular LIMO heart made from two ventricles, while excluding the valves, is approximated to be 600 to 700 ml. This falls within the range of a normal adult heart volume of 490 to 910 ml (*32*). The results demonstrate that the ventricle size required to achieve a specific SV can be adjusted by modifying the pouch size, which is ultimately constrained by factors such as the capacity of the actuation system. As shown by the results in Fig. 4C and Table 1, where we provide the same actuation pressure *P*_A_ to all prototypes, we observe that for fewer pouches, we achieve a larger maximum volumetric output *∆V*_C_, resulting in a higher EF. However, for fewer pouches, we also require considerably higher actuation volume *V*_A_.

**Table 1.** Fluidic transmission ratio *i* of LIMO ventricles with different numbers of pouches. Input volume (Δ*V*_A_), output volume (Δ*V*_C_), fluidic transmission ratio *i*, and EF of samples with different numbers of pouches against a minimum afterload of 3.8 kPa.

Sample	Input volume (Δ*V*_A_, ml)	Output volume (Δ*V*_C_, ml)	Ratio *i* at *P*_A_ = 70 kPa	EF (%)
N5	180	216	1.2	72
N7	135	195	1.44	65
N9	93	170	1.83	57
N11	83	162	1.95	54
N13	65	140	2.15	47
N15	53	116	2.19	39

These results demonstrate experimentally that all samples could reach a fluidic transmission ratio higher than one, where, for smaller and more pouches, we achieve higher ratios, reaching a maximum of 2.19 for N15 that consequently has the lowest EF (39%) among the prototypes (Table 1). It should be noted that when determining the fluidic transmission ratio, we consider the geometric volume of the pouches, not the total uncompressed volume of the air used to actuate the pouches. Effectively, we therefore assume that we are inflating the pouches using an incompressible fluid. Focusing on an SV of 90 ml that lies in the range of the SV of a healthy native heart (50 to 100 ml) (*30*), we observe that all artificial ventricles can reach this output volume, and all ventricles reach a fluidic transmission ratio higher than one except for N5 (Fig. 4C). In addition, we observe that prototypes with 11 or fewer pouches (N11, N9, N7, and N5) could reach an EF between 54 and 72% (Table 1), which equals an SV of 162 to 216 ml, against a minimum pressure of 3.8 kPa (~28 mmHg). Although the size and EF of current models are comparable to the normal values of our native ventricle (52 to 72%) (*32*, *33*), the obtained values of SV (162 to 216 ml) are notably higher than for a native heart. This gives us further opportunity to tune the design to adjust and minimize the size of the device further. It should be noted that the EF is measured against a minimum afterload of 3.8 kPa (~28 mmHg), which is comparable or slightly higher than pulmonary artery systolic pressure (*34*). Hence, for a biventricular device, distinct design parameters must be considered for each side to ensure balanced CO, as the left ventricle operates against notably higher afterloads (*31*).

We next study the afterload sensitivity of the artificial ventricle, i.e., how an increasing afterload affects the fluidic transmission ratio at given actuator pressures. To do so, we conducted similar in vitro quasi-static experiments as before while increasing the afterload (*P*_aft_) in the range of 5 to 20 kPa (37.5 to 150 mmHg) above the minimal afterload of 3.8 kPa (Fig. 4A). These are pressures in the range of those generated by a native heart. We focus on an artificial ventricle with nine pouches (N9) that we believe shows most potential for dynamic testing, as samples with fewer pouches do not reach fluidic transmission ratios that are notably higher than *i* = 1, while more pouches require higher actuation pressure and lead to narrow channels, which limit the air flow in high-frequency actuation. It should be noted that the ventricle with 11 pouches (N11) also shows similar trends.

Figure 4D shows the test results of the ventricle with nine pouches under different afterloads. We observe that the fluidic transmission ratio only starts to increase after the pressure in the pouches surpasses the pressure in the ventricle. As a result, for an increasing afterload from 5 to 20 kPa, we find that the fluidic transmission ratio at *P*_A_ = 70 kPa drops from 1.68 to 1.30. Although the afterload influences the fluidic transmission ratio at a specific actuation pressure, the pressure-volume response of the pouch actuator seems to indicate that the afterload mostly shifts the pressure, such that this effect can be circumvented by increasing the actuation pressure further to again increase the fluidic transmission ratio.

Besides providing sufficient CO, efficient transfer of energy is crucial for any transmission system. To evaluate the mechanical efficiency of work transmission in the soft artificial ventricle, we characterize the ratio of the work done by the ventricle and compare it to the input work supplied to the pouch actuator. Because mechanical work is represented by the integration of pressure over geometric volume, the input and output work is determined from the pressure-volume curves of the pouch actuators and the ventricle, respectively (fig. S5). From our analysis, we find that for a given output volume of Δ*V*_C_ = 90 ml, the mechanical efficiency increases from 82 to 91% as the *P*_aft_ increases from 5 to 20 kPa above the minimum afterload of 3.8 kPa, indicating that the LIMO ventricle transforms the energy more effectively under higher afterload. We also find that the LIMO ventricle with 9 pouches has a slightly higher efficiency than the LIMO ventricle with 11 pouches (Table 2 and fig. S5). We believe that this is because the pouch fabric undergoes stretching during actuation, consuming part of the input energy. In the LIMO ventricle with more pouches, higher pressure is required in the pouches to achieve a given output volume, resulting in greater stretching and therefore slightly more energy consumption compared to the LIMO ventricle with less pouches. Although these materials are nearly inextensible, previous modeling results still demonstrate some stretching that occurs in the pouches at these pressures (*27*).

**Table 2.** Fluidic transmission ratio *i* and energy efficiency of N9 and N11 LIMO ventricles. Measured at Δ*V*_C_ = 90 ml and mechanical efficiency of the LIMO ventricles against different *P*_aft_ above the minimum afterload.

Afterload *P*_aft_ (kPa)	Ratio *i*		Input work (N·m)		Output work (N·m)		Efficiency (%)	
	N9	N11	N9	N11	N9	N11	N9	N11
5	1.32	1.46	1.01	1.01	0.83	0.80	81.87	78.94
10	1.28	1.36	1.42	1.45	1.24	1.23	87.51	84.97
15	1.25	1.34	1.85	1.88	1.69	1.62	91.48	86.49
20	1.21	1.30	2.32	2.33	2.10	2.10	90.83	90.48

### In vitro dynamic test bench performance

**Fig. 5.**
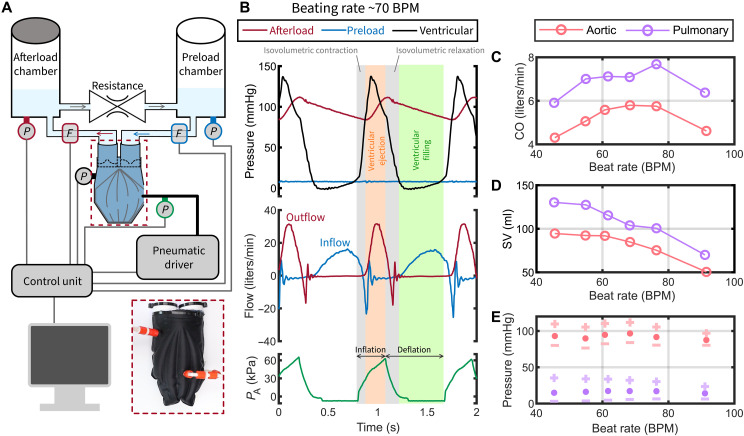
In vitro evaluation of a single ventricle in a single-sided MCL. (**A**) Single-sided MCL setup, pressure sensors (*P*), and flow sensors (*F*). (**B**) Prototype N9 functioning against aortic condition at 70 BPM. *P*_A_, actuator pressure. (**C**) Total CO from a single ventricle against aortic and pulmonary conditions. (**D**) SV at different beating rates against aortic and pulmonary conditions. (**E**) Systolic (+), diastolic (−), and average (•) pressures at different beating rates against aortic and pulmonary conditions.

To assess the behavior of the LIMO ventricle under physiological conditions and cyclic loading, we performed an in vitro experiment in a single-sided MCL (Fig. 5A). The MCL consists of two compliance chambers that mimic the afterload and preload, with a manually controlled restriction in between that is used to tune the setup for desired conditions (fig. S6). Real-time data are collected and stored using pressure sensors in the preload chamber, afterload chamber, and ventricle and using flow sensors before and after the ventricle to measure the cardiac inflow and outflow. These experiments were done to measure the generated CO by the developed prototype (N9) against aortic and pulmonary afterloads and to investigate the dynamic behavior of the system by applying cyclic loading and increasing the beating rate from 45 to 90 beats per minute (BPM) (movie S5). Both the aortic and pulmonary conditions are considered independently, to which we tune the MCL. For the aortic condition, we pump against afterloads in the range of 105 ± 6.4 to 78 ± 2.1 mmHg, respectively, as the peak systolic and end-diastolic pressures, which is characterized by a mean aortic pressure of 87.3 ± 2.6 mmHg. This is comparable to the pressures of the left side of a healthy human heart (*35*). For the pulmonary condition, we pump against a mean afterload of 14.3 ± 0.7 mmHg that is comparable to the normal working condition of the right side of the human heart, with a mean value between 11 and 17 mmHg (*36*). It should be noted that we use the mean afterload as the control parameter for MCL tuning. However, because of our MCL limitations, the peak systolic and end-diastolic pressures (31.8 ± 3.8 and 5.5 ± 1.5 mmHg) are higher and lower, respectively, than physiological levels.

Focusing first on the aortic conditions, we find that, similar to a native heart, a full cardiac cycle of the LIMO ventricle consists of four stages (Fig. 5B). A cardiac cycle starts with a very short duration of isovolumetric contraction at which the ventricular pressure (*P*_v_) is increasing without generating cardiac flow, as the afterload is higher than *P*_v_. Once *P*_v_ becomes higher than the afterload, the outlet valve opens, and fluid is pumped out of the ventricle. This causes an increase in the afterload as well. Once the inflation phase stops, *P*_v_ starts to decline rapidly, and the outlet valve closes, which results in an isovolumetric relaxation until *P*_v_ dives below the preload pressure, where the inlet valve opens and the ventricle fills up. It is important to note that the misalignment between afterload pressure and ventricular pressure occurs because the afterload pressure sensor is positioned below the afterload chamber. The distance between the afterload chamber and the LIMO ventricle introduces a delay in the peak systolic pressure of the aorta relative to ventricular pressure (Fig. 5B).

We find that, against aortic pressures, the maximum SV of 95 ml against a mean afterload of 93 mmHg occurred at the lowest beating rate of 46 BPM that we tested (Fig. 5C). In general, we found that an increase in heart rate (HR) reduces the SV. While this could be an effect of less effective pumping due to internal flow in the ventricle, we observe a decline in the maximum pressure in the pouches (table S2) by increasing the beating rate (Fig. 5C). This indicates that the driving system we used cannot supply the pressures fast enough to maintain a higher SV that is measured under quasi-static conditions. Despite that decline, we could obtain sufficient CO up to 5.9 liters/min at 70 BPM (*P*_A_ = 61 kPa), after which the CO decreases (Fig. 5D). Our LIMO ventricle could maintain mean afterloads of 88 to 97 mmHg, pumping at a rate of 4.3 to 5.9 liters/min (Fig. 5, D and E). In future work, to address the reduction in SV at higher frequencies, the actuation system should be engineered to consistently reach the desired target pressure across all operating frequencies, as our setup now did not allow to determine the effectiveness of the LIMO ventricle concept at higher frequencies. To achieve this, adopting a hydraulically driven system could provide advantages by enabling volume-controlled actuation, resulting in more precise, reliable, and consistent performance.

Next, to resemble pulmonary circulation, we set the manual valve in the single-sided MCL between the chambers to its fully open position. Nevertheless, we observe that the resistance is still slightly high as the peak systolic pressure goes above 30 mmHg. Despite higher resistance, we were able to achieve a maximum CO of 7.6 liters/min at 76 BPM (*P*_A_ = 39 kPa). Our LIMO ventricle could maintain mean afterloads of 14 to 17.3 mmHg, pumping at a rate of 6 to 7.6 liters/min (Fig. 5D). It should be noted that the actuator pressure was reduced to 50 kPa in this setting, because the afterload is also lower than the aortic condition and we wanted to avoid overloading the system. Despite that, we observed that the CO in the pulmonary condition is still higher than the CO against the aortic condition.

## DISCUSSION

In this study, we proposed a fluidic transmission system with volume output/input ratios that exceed current existing solutions that are inherently limited because of their membrane-based design. We demonstrated that in our system that showed relatively low energy losses (Table 2), smaller actuator volumes at higher pressures are transformed to larger volumes at lower pressures (Fig. 4C and Table 2). This characteristic is essential in the next generation of fluidically driven TAHs, where the size of the device can be reduced to enable full implantation of both the artificial heart and the systems required to control and power it.

Overall, the fluidic transmission system with nine pouches around its circumference has an energy transfer between 82 and 91%, which means that less than 1.22 W of net fluidic input power needs to be supplied to the system to output 1 W of fluidic power to the blood, corresponding to a CO = 5 liters/min, against a mean afterload of 90 mmHg. Note that this is a lower bound on the energy input, because there are necessarily additional losses in the driving fluid supply system, including viscous losses and efficiency losses of the driving pump. Still, this is a promising energy transfer efficiency that is needed in the development of a fully implantable control and battery system in the future.

Moreover, while here we only validated a single ventricle at both aortic and pulmonary conditions, we did not test our LIMO heart with two ventricles in a double-sided MCL. At the moment, a full TAH could, for example, be obtained using two separate ventricles, actuated either simultaneously, as in the SynCardia design, or alternately, as in the Carmat system. It is too early to conclude what the best approach is for our LIMO concept. We hypothesize that in the case of simultaneous actuation, which mirrors the function of the native heart, integrating both ventricles into a single structure could enhance the fluidic transmission ratio, as a portion of the pouches can be incorporated into the shared septal wall to assist in contracting both the left and right sides of the TAH. However, this configuration would require an additional volume to store the driving fluid during diastole. When considering alternating actuation, an additional volume is not necessary, allowing for a more compact system. The trade-off, however, is that a larger volume must be displaced in each cycle, compared to the simultaneous actuation approach. Future work will focus on the development of a biventricular device and the optimization of its actuation mechanism, taking into account these considerations to achieve an optimal balance between system size, efficiency, and physiological performance.

Moreover, each side of our native heart is pumping against different pressures. In developing a TAH with two ventricles, one of the primary challenges will be to ensure a balanced output between the left and right ventricles, which is a vital factor for the successful mimicry of the heart’s natural mechanical function. Toward that challenge, we demonstrated that by tuning the design of pouch geometries, we can achieve different COs against similar afterload or similar CO against different afterloads. It enables us, in future studies on biventricular TAH development, to optimize the geometry of pouches and ventricles, to obtain equal CO despite their different afterloads. In addition, an important consideration for a TAH is that it must dynamically adjust ventricular output in response to changes in the circulatory system to maintain healthy blood pressure. This requires each ventricle to exhibit preload sensitivity, which underlies the Frank-Starling mechanisms that describe the ability to accommodate increased filling volume without a notable rise in pressure when unactuated. Soft materials and structures inherently support this functionality. We already observe preload sensitivity in our samples (fig. S5, B and E), although we did not specifically design our ventricle to demonstrate this effect. Yet, further design optimization should be used when working toward a biventricular device. Further enhancement of this feature could be achieved through improved design and the use of more stretchable fabrics, which will be essential in future developments.

While this study demonstrates the proof of concept for our efficient fluidic transmission system, we acknowledge several directions that need to be further investigated to advance this technology toward TAH development. First, the proposed fabrication method and materials are optimized for cost-effective and rapid prototyping, enabling the essential experiments conducted in this study. However, to ensure long-term durability, further advancements in both materials and fabrication techniques are necessary, as the system must withstand millions of cycles over a lifetime of operation. Second, biocompatibility was not considered in this study, yet it is a critical factor influencing both material selection and device design. Future research should address these aspects to ensure safe and reliable long-term implantation. In addition, pouch configuration should be further investigated to optimize flow patterns, minimizing stagnation points and reducing the risk of thrombosis. Third, in our experiments, the device was pneumatically actuated using air, which is not a viable option for an implantable device due to safety concerns and air compressibility limitations that restrict higher COs at increased frequencies. Future iterations should incorporate a hydraulic actuation system, which would not only improve control reliability but also enable volume-controlled actuation for more precise performance. Last, our MCL has limited capabilities in replicating a wide range of hemodynamic conditions, which are essential for evaluating a TAH. Therefore, future studies on a biventricular device should be conducted using a more advanced MCL, capable of simulating both healthy and pathological conditions for comprehensive evaluation.

## MATERIALS AND METHODS

### Design and fabrication

To mimic the natural movements and characteristics of a human heart more closely, we design and fabricate a soft artificial ventricle. In this concept, we use parallel cylindrical pouches along the height of the ventricle as soft artificial muscles. The pouches inflate and deflate using a pneumatic driving system that provides positive and negative pressure during respectively inflation and deflation. To construct the pouches, we use a heat sealing technique to bond two layers of thermoplastic sheets together using specific patterns designed in 2D. For this purpose, we customized a 3D CNC machine (TEC4, FELIX, IJsselstein, The Netherlands) by adding a hot rolling tip as the end effector. Using the G-code generated for a 2D pattern, the machine moves the hot tip on sheets at a low speed (200 mm/min) to seal them together. The nozzle and bed temperatures are set to 275° and 70°C, respectively, to seal two layers of TPU-coated nylon (Riverseal 70 LW, 78Dtex; 170 g/m^2^; Rivertex, Culemborg, The Netherlands). It is a nearly inextensible material yet flexible and thin. The 2D patterns are designed in Adobe illustrator software (Adobe Illustrator, Adobe, California, United States) (fig. S1A). In our design, all pouches are placed within a frame and separated from each other by a sealing line. There is a gap between each sealing line to let the air reach all the pouches. As a result, we do not need a separate opening for each pouch, and they are all connected (fig. S1). After that, we cut the leftovers from only one side of the prepared pouches, fold the prepared pouch arrays along the middle line, and then do another heat sealing using a 2D pattern that creates the ventricle chamber. We leave a single opening at the top of the ventricle that is filled with a 3D-printed TPU part, which holds two mechanical heart valve prostheses with opposing orientations, creating an inlet and an outlet (Fig. 3 and fig. S1B). In a variation on this design, we created ventricles with two smaller openings (fig. S1C), and we used this design in our quasi-static experiments, where we connected the ventricle to the water chamber with a single port used for both the inlet and outlet. In both cases, we made a hole in one pouch on each side of the ventricle to insert 3D-printed soft TPU connectors that are used for the air inlet and outlet. A larger 3D-printed soft TPU connector is also placed in the ventricle wall to measure ventricular pressure (Fig. 3 and movie S2).

### Blocked displacement testing of pouch arrays

For our cylindrical model, if we make a cut from one of the seam lines and unwrap the ventricle, then we will have an array of pouches that is known as a soft actuator called pouch motors (*26*). To explore the possibility of getting the same result in various designs, we performed a blocked displacement experiment on four pouch arrays (N4, N8, N10, and N20) with different numbers of pouches (4, 8, 10, and 20), each with a constant total height of *H* = 20 cm. All samples have a width of *W* = 6 cm. The total height of the pouch arrays is divided equally between the pouches, and the pouches are separated by seal lines (seams) of ~2 mm wide. Therefore, a single pouch has a height of 48, 23, 18, and 8 mm, respectively. Samples are made by heat sealing two layers of TPU-coated nylon (Riverseal 70 LW, 78Dtex; 170 g/m^2^; Rivertex, Culemborg, The Netherlands) together using a 2D pattern designed in a sketching software (Adobe Illustrator, Adobe, California, United States) (fig. S2).

Samples are then clamped in a tensile testing machine (INSTRON 5965, Norwood, United States). The initial length between the top and bottom clamps is set to 20 cm, equal to the total height of the samples. Before pressurizing the pouches, we apply a relatively low negative pressure of −5 kPa to ensure that the initial geometric volume of the pouches is zero. Then, the pouches are pressurized up to 4 bars in 5 min at a constant rate, after which the pressure is reduced back to −5 kPa at the same rate. Meanwhile, the force applied to the jaws is measured using the INSTRON machine (movie S2). We refer to this experiment as a blocked displacement experiment (*27*). Thus, the jaws do not move during the test. The air pressure and air flow are measured using respectively an air pressure sensor (MPX5100DP, NXP, Eindhoven, The Netherlands) and a low-range flow sensor (HAFBLF0750CAAX5, Honeywell, North Carolina, United States). Geometrical volume change (*∆V*_A_) of the samples is calculated using pressure and mass flow data obtained by the sensors, considering the ideal gas law.

### In vitro quasi-static characterization against no afterload

Although we could assess our concept theoretically by the proposed model, there are some factors affecting the behavior of the device that are not considered in the model, such as the exact geometry and the deformation in the material due to its elasticity. Therefore, to explore the behavior of the system in a physical setup and to proof the fluidic transmission concept experimentally, an in vitro setup was designed and built, enabling us to perform quasi-static experiments to evaluate and characterize the prototypes. The setup consists of a chamber containing colored water. A pressure sensor (RS PRO 828-5726, London, England, UK) is placed below the chamber to measure the pressure of the water column, by which we could also measure the volume displaced from the prototypes to the chamber, extracting the volume of the chamber.

The aim of this experiment was to assess the behavior of samples with different numbers of channels against almost no afterload. Thus, no air pressure is applied to the top of the chamber to simulate afterload, and the only minimum afterload that is applied to the system is caused by the height of the water column. A digital pressure controller (VEAB-L-26-D13-Q4-V1-1R1, Festo, Esslingen am Neckar, Germany) is used to actuate the samples. Six samples underwent this experiment; so-called N5, N7, N9, N11, N13, and N15 with 5, 7, 9, 11, 13, and 15 pouches distributed, respectively, around the circumference of the prototype (figs. S1 and S3). The prototypes have an equal initial circumference of 22 cm. Therefore, the higher the number of pouches, the smaller the size of each individual pouch. The samples that are tested in the quasi-static setup have a single opening that is connected to the bottom of the water chamber, through which water flows into and out from ventricle. To run the system, we slowly increase the pressure linearly from −5 to 70 kPa in 90 s (0.83 kPa/s) to make sure that there are no dynamic effects. The pressure inside the pouches is measured by a pressure sensor (MPX5100DP, NXP, Eindhoven, The Netherlands) during the cycle. To measure the volume displacement of the pouches, we used a one-directional thermal mass flow sensor (AWM5101VN, Honeywell, North Carolina, United States), measuring the mass of air into and out from the pouches. To use only one sensor for measuring both inflow and outflow, we put it in a circuit, by which we could change the flow direction using manual valves, so that the flow is always passing through the flow sensor in the same direction, whether it is inflow or outflow (fig. S4). We wait 10 s between each step to let the system settle and to change the flow direction. Volume displacement of the pouches is then calculated by the pressure and mass flow, considering the ideal gas law.

### In vitro quasi-static characterization against physiological afterloads

To evaluate the prototypes working against afterloads comparable to physiological pressures, two of the samples (N9 and N11) were chosen to undergo quasi-static experiments with applied afterload above the water chamber. In this experiment, the chamber was closed off, and the air pressure above the chamber was set and controlled by a digital pressure controller (VEAB-L-26-D13-Q4-V1-1R1, Festo, Esslingen am Neckar, Germany) and monitored by a pressure sensor (RS PRO 828-5726, London, England, UK). The pressure controller was used to maintain the pressure above the water constant during each test. The samples were tested four times against 5, 10, 15, and 20 kPa, respectively. It should be noted that these are values to which we set the pressure controller, above the minimum pressure of 3.8 kPa. Thus, the pressure inside the ventricle is slightly higher than that of the water height. We used the data from the pressure sensors (RS PRO 828-5726, London, England, UK) on the top and bottom of the chamber to calculate water displacement.

A full actuation cycle consists of inflating the pouches from −5 to 70 kPa linearly in 90 s (0.83 kPa/s) by a proportional pressure controller (VEAB-L-26-D13-Q4-V1-1R1, Festo, Esslingen am Neckar, Germany), then waiting for 10 s to settle, and deflating the pouches back to −5 kPa. We repeat each cycle three times against each afterload condition. We used a thermal mass flow sensor (AWM5101VN, Honeywell, North Carolina, United States) and a differential pressure sensor (MPX5100DP, NXP, Eindhoven, The Netherlands) to characterize the flow and pressure in the pouch, respectively. The geometric volume of the pouch actuator $V_{A}$ was calculated by

$$V_{A}=\frac{P_{\text{atm}}V_{p\_0}}{P_{A}+P_{\text{atm}}}+\frac{\int Qdt\cdot T_{\text{room}}}{P_{A}+P_{\text{atm}}}\cdot \frac{P_{\text{atm}}}{T_{\text{zero}}}$$ (4)

where Q  is the mass flow rate (at standard temperature and pressure) measured by the flow sensor, $P_{A}\;$  is the pouch actuator pressure (relative to atmosphere pressure $P_{\text{atm}}$ ) measured by the pressure sensor, $V_{P\_0}$ is the initial geometric volume of the pouch at atmosphere pressure, and $T_{\text{room}}=293\;K$ and $T_{\text{zero}}=273\;K$ represent the room and zero temperatures, respectively. We assume that $V_{P\_0}=0$ because the pouch was vacuumed at −5 kPa in each test. Therefore, the geometric volume change of the pouch was calculated by

$$∆V_{A}=V_{A}-V_{P\_0}=V_{A}$$ (5)

We used a pressure sensor (RS PRO 828-5726, London, England, UK) to measure the water pressure inside the ventricle $P_{C}$ (relative to atmosphere pressure). We placed two pressure sensors (RS PRO 828-5726, London, England, UK) at the top and bottom of the water cylinder, respectively, to measure the change of the height of the water column $∆H_{\text{water}}$ . The geometric volume change of the ventricle $∆V_{C}$ was calculated by

$$∆V_{C}=\pi R^{2}∆H_{\text{water}}$$ (6)

where R is the radius of the water cylinder. The input work done by the air in the pouch can be calculated by

$$W_{\text{in}}=\int P_{A}d∆V_{A}$$ (7)

Note that we assume that the initial work done by loading the ventricle is negligible compared to the input work done by the pouch actuator. The output work done on the ventricle can be calculated by

$$W_{\text{out}}=\int P_{C}d∆V_{C}$$ (8)

The mechanical efficiency of the LIMO heart is defined as

$$\eta =\frac{W_{\text{out}}}{W_{\text{in}}}\times 100\%$$ (9)

Figure S5 shows the test results of the artificial ventricles with 9 and 11 pouches, respectively. The input work is calculated by eq. S4, which corresponds to the area under the pressure-volume curves of the pouch actuator (fig. S5, A and D). The output work is calculated by eq. S5, which corresponds to the area under the pressure-volume curves of the ventricle (fig. S5, B and E). The mechanical efficiencies of N9 and N11 ventricles are calculated by eq. S6 at various afterloads (fig. S5, C and F). Both N9 and N11 ventricles show higher mechanical efficiency with increasing afterloads. The N9 ventricle shows slightly higher efficiencies than the N11 ventricle. Note that the rise and fall in the pressure-volume curves of the ventricle in fig. S5 (B and E) are due to the flow resistance present in the experimental setup.

### In vitro evaluation in a dynamic test bench

#### Actuation method

The pouches are actuated pneumatically using a digital pressure controller (VPPE-3-1-1/8-2-010-E1, Festo, Esslingen am Neckar, Germany) in a pulsatile manner. Their inflation induces systole, followed by their deflation to create diastole. The HR is determined by the timing of systolic (*t*_sys_) and diastolic (*t*_dia_) phase. To drive the pouches, two digital solenoid valves (MHE2-MS1H-5/2-QS-4-K, Festo, Esslingen am Neckar, Germany) connect them to either a pressure containing pressurized air at a fixed pressure, adjusted by the pressure regulator, or a tank with fixed negative pressure (−7 kPa) generated by a vacuum pump (VN-14-L-T4-PQ2- VQ3-RO2, Festo, Esslingen am Neckar, Germany). Inflation occurs when the pouches are linked to pressurized air, and deflation occurs when the pouches are connected to the vacuum tank. Control over the solenoid valves allows for adjustments in the beating frequency and the timing of systolic and diastolic phase, facilitating the exploration of the LIMO heart’s performance under various conditions. The maximum pressure inside the pressure tank in these experiments is set to 70 and 50 kPa, when working against aortic and pulmonary conditions, respectively. However, we observed that the pouch actuator pressure does not always reach the set value due to the circuit resistance at elevated actuation frequencies. This results in a reduction in SV for increasing the beating rate, which is not intrinsic to the LIMO heart design but is a result of the LIMO heart integrated with its pneumatic control system.

#### Mock circulatory loop

The prototype N9 was tested in vitro in a single-sided MCL designed according to the Windkessel model (fig. S6). Our MCL replicates various aortic and pulmonary afterloads and preloads, along with peripheral resistances and compliances for both conditions. The systemic and pulmonary arterial circulations (afterloads) are modeled with a two-element Windkessel consisting of a compliance chamber followed by a manually adjustable resistance valve (type 3232 2/2 way diaphragm valve, Burkert, Ingelfingen, Germany). The compliance in these chambers can be adjusted by changing the water level. A resistance valve is used to replicate vascular resistance for both pulmonary and aortic conditions separately. Venous compliances and preloads for the right or left sides are represented using a chamber open to air, where water levels are adjustable. Pressure under both preload and afterload chambers is monitored by electronic pressure sensors placed under each chamber (RS PRO 828-5726, London, England, UK). Cardiac outflow and inflow are measured using ultrasonic flow sensors (DIGIFLOW-EXT1, Emtec, Finning, Germany) that are clamped on hoses (Tygon formula E-3603 laboratory tubing Z765139, Saint Gobain, La Défense, France) coming out of the device. For in vitro testing, tap water was used. All sensor data were captured using a data acquisition card (USB-6218, National Instruments, Austin, Texas, United States).

We tested the N9 prototype under pulmonary and aortic conditions at frequencies set to 50 to 100 BPM in steps of 10. Inflation time (*t*_sys_) and deflation time (*t*_dia_) are set approximately to one-third and two-thirds of the whole cycle duration, respectively. Note that the actual beating rate is less than that of what we set due to a delay caused by the software in compiling the driving script line by line (table S1).

To analyze the experimental data obtained from the MCL experiment and to determine the actual beating rate, SV, and total CO, a set of data including 5 consecutive cycles is taken out from the steady-state working period that is reached after adjusting the MCL. The average SV ( ${\text{SV}}_{\text{avg}}$ ) is measured by integrating the outflow (*Q*_water_) curves of these 5 cycles during systole phase and averaging them. However, for some of the experiments, we had to use inflow curves during diastole to measure SV, because the peak outflow was higher than the normal measuring range (up to 32 liters/min) of our sensors.

$$\text{SV}=\int Q_{\text{water}}dt$$ (10)

HR is determined by dividing the total time of these cycles by five, and then the total CO is calculated by

$$\text{CO}=\text{HR}\times {\text{SV}}_{\text{avg}}$$ (11)

## Supplementary Materials

### The PDF file includes:

Figs. S1 to S10 Tables S1 to S3 Supplementary Text Legends for movies S1 to S5 References
