# Supplementary material for: Toward developing a compact total artificial heart using a soft robotic fluidic transmission system
Source: Sci Adv. 2025 Jul 2;11(27):eadv4854. doi: 10.1126/sciadv.adv4854 (PMC12219477; doi:10.1126/sciadv.adv4854)
Supplement: Supplementary file 1 — Figs. S1 to S10 Tables S1 to S3 Supplementary Text Legends for movies S1 to S5 References [file sciadv.adv4854_sm.pdf]

Science Advances
AAAS

Supplementary Materials for

Toward developing a compact total artificial heart using a soft robotic fluidic transmission system

Maziar Arfaee *et al.*

Corresponding author: Johannes T. B. Overvelde, b.overvelde@amolf.nl

Sci. Adv. **11**, eadv4854 (2025)
DOI: 10.1126/sciadv.adv4854

The PDF file includes:

Figs. S1 to S10
Tables S1 to S3
Supplementary Text
Legends for movies S1 to S5
References

Other Supplementary Material for this manuscript includes the following:

Movies S1 to S5

Supplementary materials

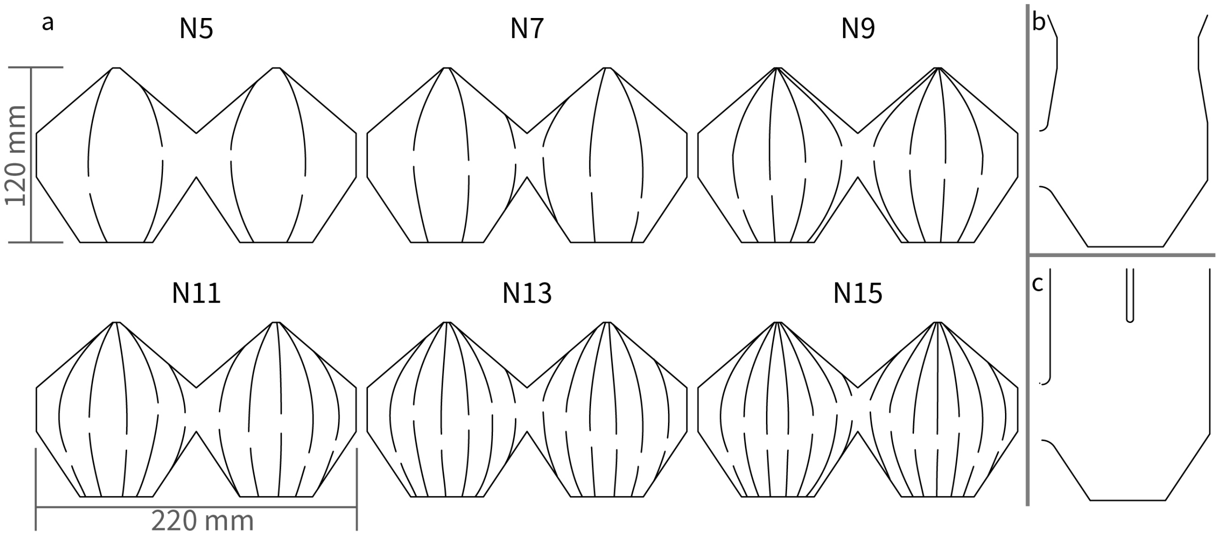

Figure S1. 2D pattern of pouches of LIMO ventricles. a) 2D heat-sealing pattern of pouches of the artificial ventricles with different number of channels, ranging from 5 to 15. b) 2D heat-sealing pattern of the second sealing step to create a single-opening ventricle. c) 2D heat-sealing pattern of dual-opening ventricle.

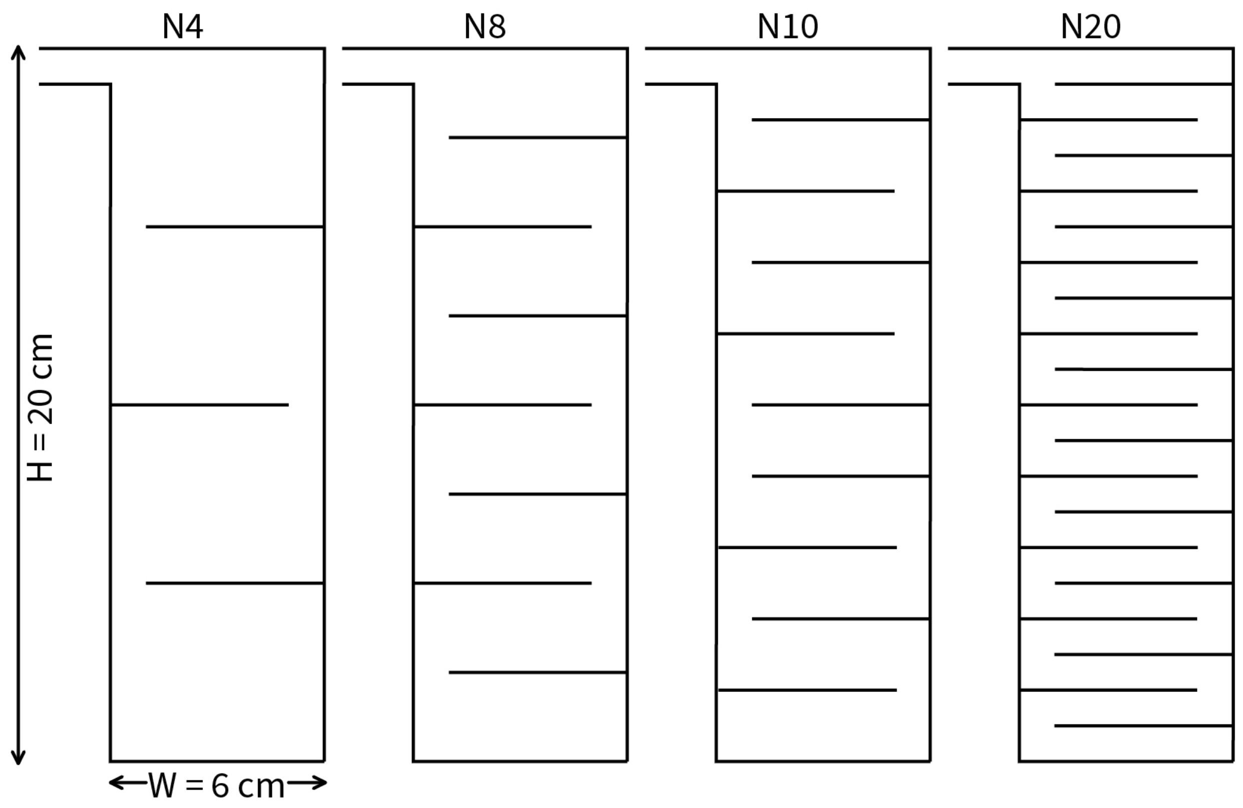

Figure S2. 2D pattern of pouch arrays. These patterns are used for heat-sealing and creating pouch motor samples.

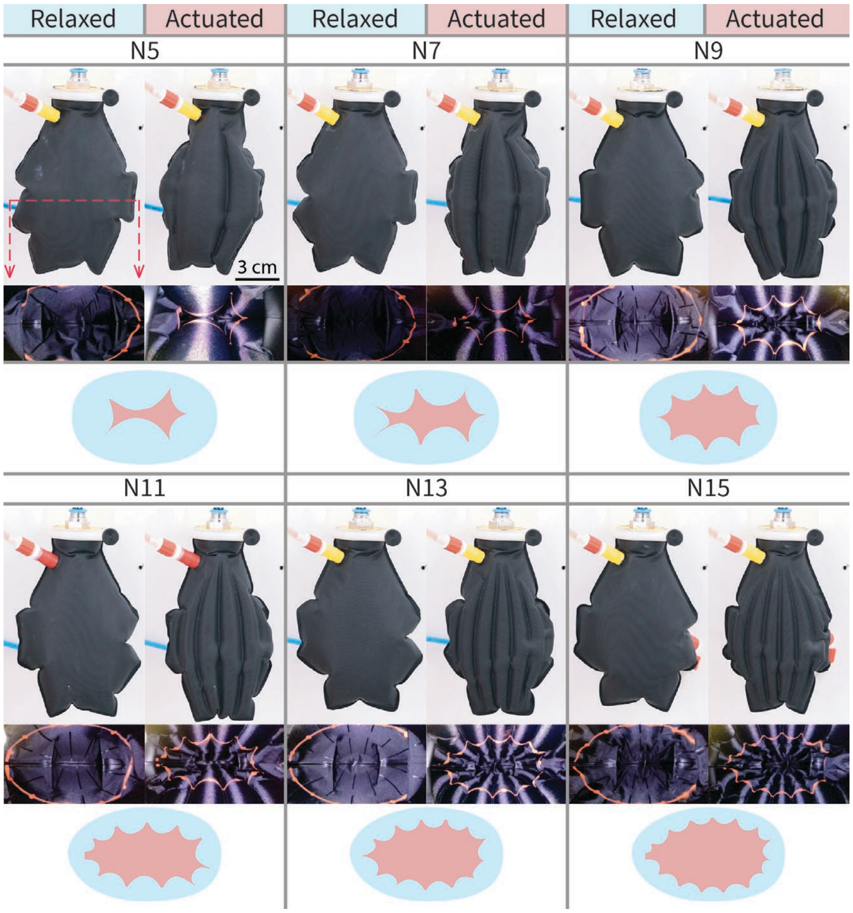

Figure S3. Relaxed and actuated states of prototypes with different number of pouches. Demonstrating the difference in intraventricular deformation resulting in various fluidic transmission ratios i and ejection fractions EF. Intraventricular deformations are shown at maximum actuator pressure of P_A = 40 kPa against identical internal pressure of P_C = 10 kPa; light blue: inner surface area at relaxed state, light red: inner surface area at actuated state.

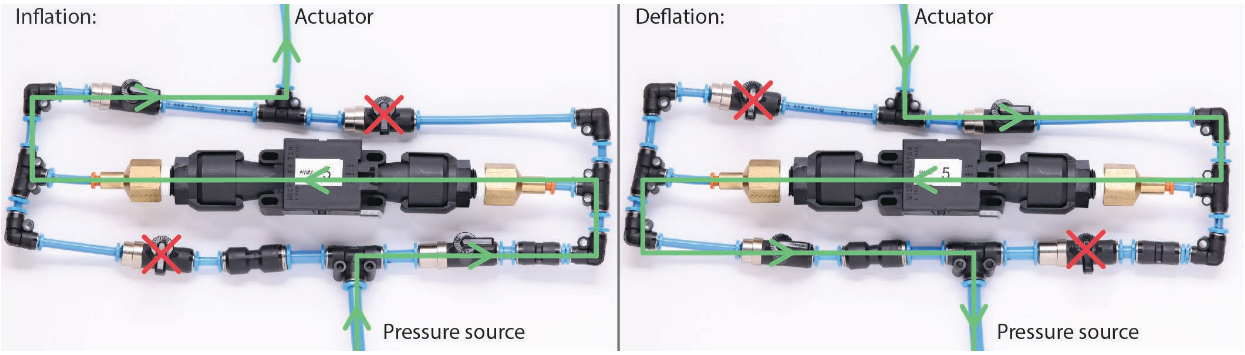

Figure S4. Volume change measurement. Mass flow sensor circuit configuration at inflation and deflation phase.

In vitro quasi-static characterization against physiological afterloads

Figure S5 shows the test results of the artificial ventricles with 9 pouches and 11 pouches, respectively. The input work is calculated by equation (4), which corresponds to the area under the pressure-volume curves of the pouch actuator (Figure S5.a, d). The output work is calculated by equation (5), which corresponds to the area under the pressure-volume curves of the ventricle (Figure S5.b, e). The mechanical efficiencies of N9 and N11 ventricle are calculated by equation (6) at various afterloads (Figure S5.c, f). Both N9 and N11 ventricles show higher mechanical efficiency with increasing afterloads. The N9 ventricle shows slightly higher efficiencies than the N11 ventricle. Note that the rise and fall in the pressure-volume curves of the ventricle in figure S5.b and e are due to the flow resistance present in the experimental setup.

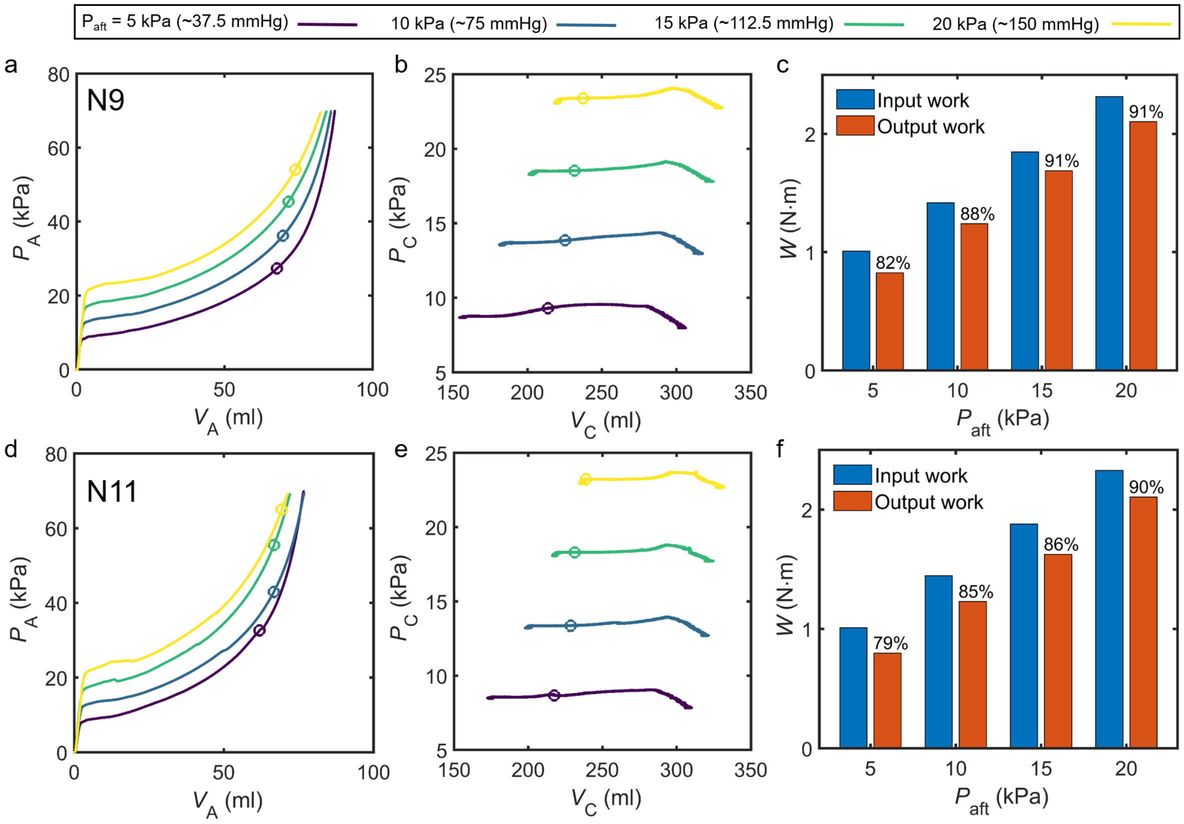

Figure S5. In vitro quasi-static test results. a, b, c) The results of N9 LIMO ventricle against physiological afterloads. d, e, f) The results of N11 LIMO ventricle against physiological afterloads. P_A : Actuator pressure, V_A : Actuator volume, P_C : Ventricular pressure, V_C : Ventricular volume.

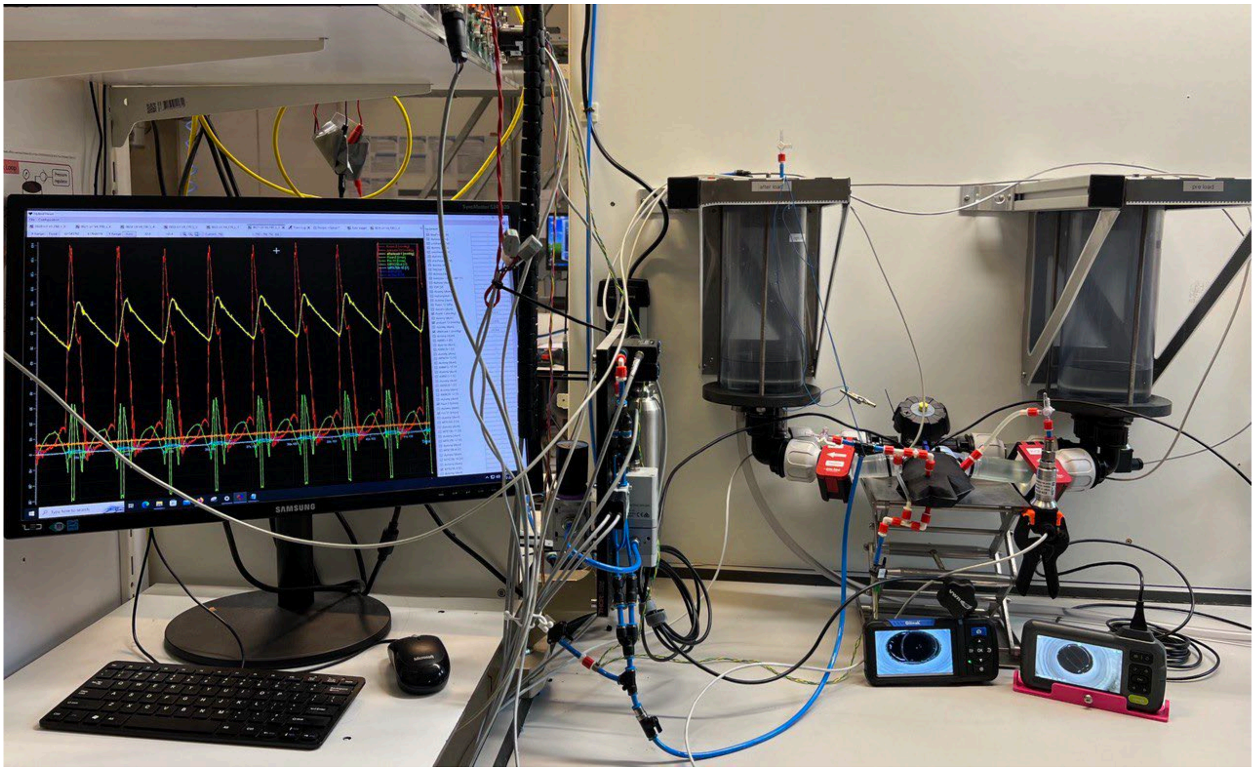

Figure S6. The single-sided mock circulatory loop. The setup used for testing the dynamic performance of the N9 prototype.

Table S1. Dynamic test condition. Set and actual beat rate per minute (BPM), and the maximum pressure reached against each condition.

Beating rate (BPM)		Maximum pouch actuator pressure (kPa)	
~set	~actual	Pulmonary (set to 50 kPa)	Aortic (set to 70 kPa)
50	46	50.2	71.7
60	55	48.6	67.9
70	61	46.6	61.9
80	69	42.7	63
90	75	38.8	56
100	91	31.8	46.4

Analytical model, effect of seam width and number of pouches

Our simplified analytical model assumes an initial cylindrical shape of the ventricle with internal volume V_0, and N pouches of length L_0, equally distributed around the perimeter and separated by seams of width s. Since the material is assumed to be inextensible, the pouch opening angle $0 \leq \theta \leq \pi/2$ fully describes the system's deformed shape. Here, we expand on the key results described in the main text, equations (1) – (3) and figure 1. Specifically, we provide analytical expressions for the fluidic transmission ratio i and ejection fraction EF at full actuation ($\theta = \pi/2$),

$$i_{\theta=\pi/2} = \frac{2\,N\,s_{\mathrm{L}} - 2\,N + \frac{N\,\pi^2}{2} - s_{\mathrm{L}}\,\pi^2 + \pi^2 - 2\,\pi\,N\,s_{\mathrm{L}} + \frac{N\,s_{\mathrm{L}}\,\pi^2}{2}}{2\,\pi^2\,(1 - s_{\mathrm{L}})}, \tag{S1}$$

$$\mathrm{EF}_{\theta=\pi/2} = \frac{2\,(1 - s_{\mathrm{L}})\left(2\,N\,s_{\mathrm{L}} - 2\,N + \frac{N\,\pi^2}{2} - s_{\mathrm{L}}\,\pi^2 + \pi^2 - 2\,\pi\,N\,s_{\mathrm{L}} + \frac{N\,s_{\mathrm{L}}\,\pi^2}{2}\right)}{N\,\pi^2} \times 100\%, \tag{S2}$$

where we define dimensionless seam width $s_{\mathrm{L}} = s / (s + L_0)$, $0 \leq s_{\mathrm{L}} \leq 1$.

These expressions are further simplified when we assume seam width is negligeable ($s_{\mathrm{L}} = 0$),

$$i_{\theta=\pi/2, s_L=0} = \frac{\frac{N\,\pi^2}{2} - 2\,N + \pi^2}{2\,\pi^2}, \tag{S3}$$

$$\mathrm{EF}_{\theta=\pi/2, s_L=0} = \frac{2\left(\frac{N\,\pi^2}{2} - 2\,N + \pi^2\right)}{N\,\pi^2} \times 100\%, \tag{S4}$$

from which we arrive at the conclusions about i and EF that we present in the main text,

$$\lim_{N\to\infty} i_{\theta=\pi/2, s_L=0} = \infty, \tag{S5}$$

$$\lim_{N\to\infty} \mathrm{EF}_{\theta=\pi/2, s_L=0} = \left(1 - \frac{4}{\pi^2}\right) \times 100\%. \tag{S6}$$

We illustrate the modeled behavior in figure S7. For non-negligible seam width, as s_{L} increases, seam width s increases at the expense of pouch length L_0. Interestingly, selecting relatively wider

seams increases i (and decreases EF), as does increasing the number of pouches N (Figure S7.a and b). However, the asymptotic behavior differs significantly. Ever wider seams ultimately result in a fully passive ventricle, such that the ejection fraction vanishes (Figure S7.c). In contrast, for negligeable seam width, increasing the number of pouches results in a linear increase of the fluidic transmission ratio i, while the ejection fraction EF approaches 59.5% (Figure S7.d).

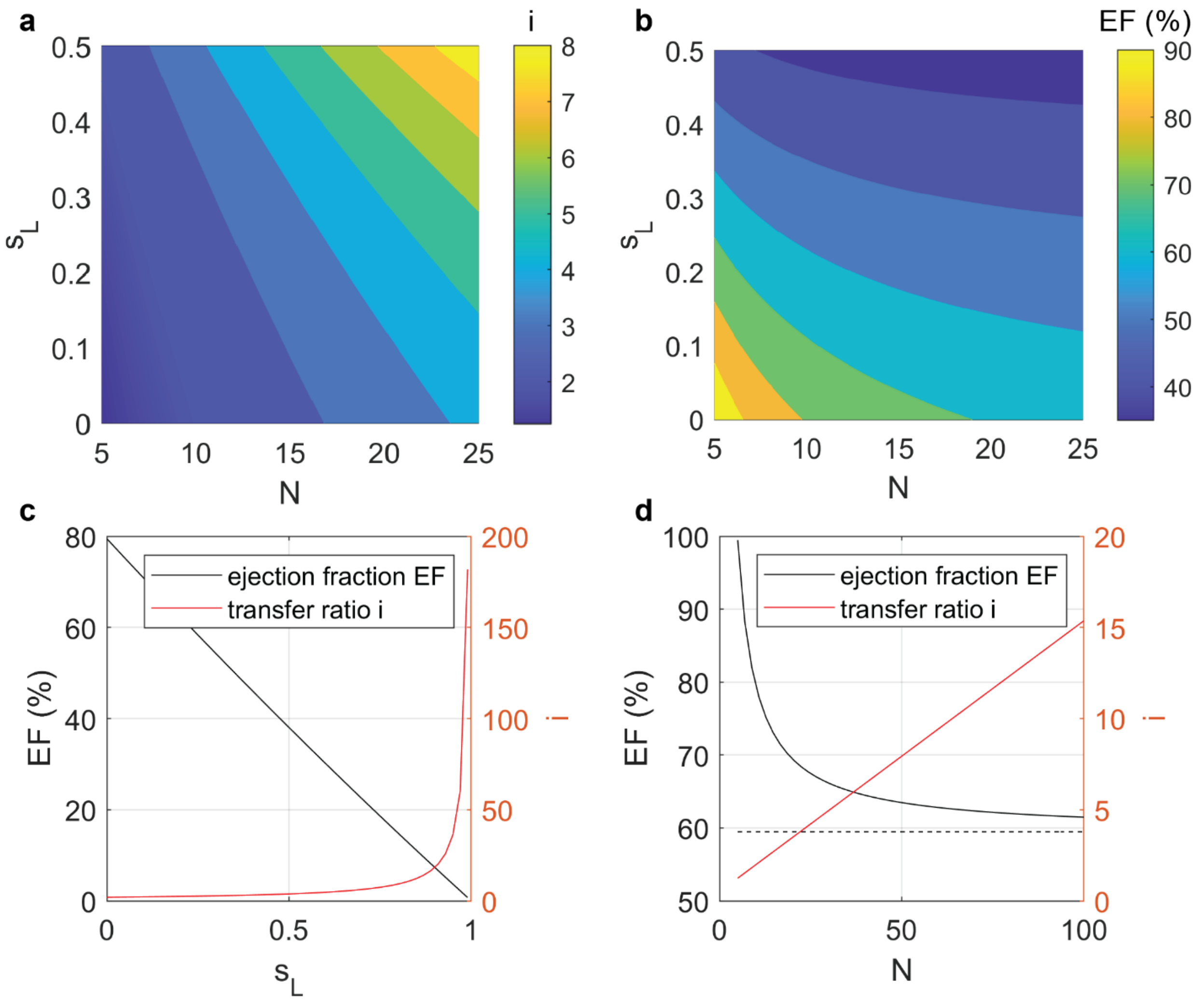

Figure S7. Idealized model-based effect of number of pouches N and dimensionless seam width sL. a) Effect on fluidic transmission ratio i. b) Effect on ejection fraction (EF). c) Effect of s_L for $N = 10$. d) Effect of N for $s_L = 0.01$, dashed line indicates asymptotic value EF = 59.5%.

Blocked-displacement testing of pouch arrays

In these experiments, samples N4, N8, and N10 broke before reaching the maximum set pressure of 400 kPa, at respectively 159.5, 249.9, and 241.3 kPa (P_{max}). The samples generated maximum force (F_{max}) of 319.7, 310.2, and 270.1 N, respectively. Sample N20 did not fail and could generate the maximum force of 208.3 N at an actuator pressure of 398 kPa (Table S2). As predicted, by increasing the number of the pouches, i.e., by reducing the size of the individual pouches, the pressure required to reach a certain force increases while the geometric volume reduces. We observed a general trend where smaller pouches typically withstand higher pressures than larger pouches (Figure S8). However, among samples with relatively minor differences in pouch size, imperfections caused by our heat-sealing technique could cause failure points that deviate slightly from this trend, as all samples ruptured near the sealing lines.

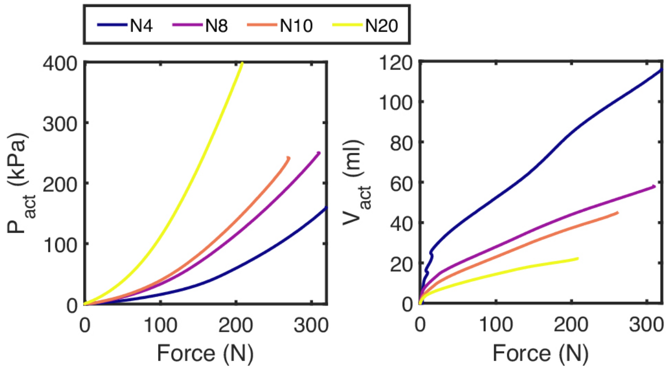

Figure S8. Force response of pouch arrays with 4, 8, 10, and 20 pouches. Left: Force-Actuator pressure (F-P_{act}). Right: Force-Actuator volume (F-V_{act}).

Table S2. Pressure, volume and generated force of each pouch array just before failure.

Sample	P_{max} before failure (kPa)	V_{max} before failure (mL)	F_{max} before failure (N)
N4	159.5	116	319.7
N8	249.9	57.9	310.2
N10	224.2	45.1	261.6
N20*	398.8	22.2	208.3

*N20 did not fail, and survived the whole experiment.

Fully soft prototype utilizing 3D-printed prosthetic valves as inlet and outlet

Although mechanical heart valve prostheses are reliable and being used as a therapy for heart valve treatments, they require lifelong management with anticoagulants and regular follow-up to monitor for complications that mainly concern their biocompatibility [37]. In this study, we used mechanical heart valve prostheses in our LIMO heart as a practical and repeatable solution suitable for *in vitro* trials. To show the feasibility of making a fully soft LIMO heart, we made a prototype by employing two 3D-printed prosthetic valves for inlet and outlet (Figure S9.a). The valves have an inner diameter of 30 millimeter and coaptation height of 5 millimeters to avoid any back flow [38]. They are printed from thermoplastic polyurethane (TPU) shore 60A filament using a FDM 3D-printer (TEC4, FELIX, IJsselstein, the Netherlands). For this prototype, we fabricated the ventricle using the sealing pattern shown in figure S1.c. It has two openings that hold inlet and outlet valves. Interestingly, this prototype could deliver maximum cardiac output CO = 8.8 l/min at 81 BPM against pulmonary condition (35/9 mmHg). However, it failed working against aortic condition (Figure S9.b). The rupture happened at the bottom of U-shape sealing path that separates the inlet and outlet (Figure S1.c, S9.c) that indicates the high stress concentration at that point. It should be noted that the sealing lines are more generally weak points of the samples, as the sheets are heated up to 275 °C and compressed. A full summary of stroke volume and cardiac output of both models against aortic and pulmonary settings at various beating rates is presented in table S3. Using a single housing for both inlet and outlet valves seems to constrain the ventricle, leading to a reduction in SV resulting in 1.2 l/min reduction in total cardiac output. Future studies need to be performed to optimize the design to maximize the cardiac output as well as improving durability by reducing stress concentrations.

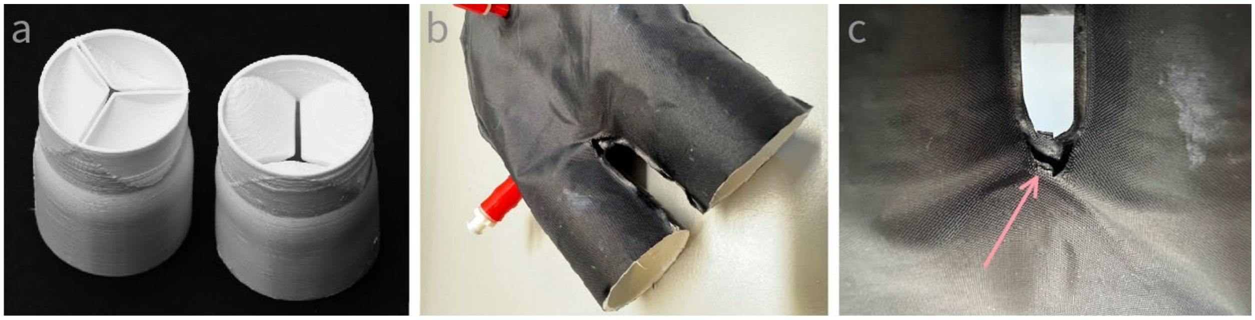

Figure S9. Fully soft prototype. a) Inlet and outlet valves 3D-printed from TPU. b) Placement of the valves in two separate openings. c) Rupture at the bottom of the U-shaped sealing line that separates the inlet and outlet valves

Table S3. In vitro results of N9 LIMO ventricle in dynamic test bench. Stroke volume (SV) and cardiac output (CO) of LIMO heart and fully soft LIMO heart against pulmonary and aortic conditions at various beating rates (HR).

		HR (BPM)	SV (mL)	CO (l/min)
LIMO heart with mechanical heart valves	Pulmonary	46	130.3	6
		55	127.5	7
		62	115.4	7.2
		69	103.9	7.2
		76	100.6	7.6
		91	70	6.4
	Aortic	45	94.5	4.3
		55	92.3	5.1
		61	91.9	5.6
		70	84.8	5.9
		76	75.3	5.7
		91	50.5	4.6
Fully soft LIMO heart	Pulmonary	46	135.6	6.3
		55	130.6	7.2
		64	124.9	8.1
		73	113.7	8.3
		81	108.6	8.8
		91	89	8.1

Life-time evaluation of current LIMO heart prototype

Each sample tested in the mock circulation loop (MCL) under short-term dynamic conditions was able to operate for approximately 1,500–2,000 cycles before failure. However, since the experiments were not conducted continuously, this does not provide a realistic estimate of the actual lifetime, as fatigue effects are largely overlooked in short-term testing. To assess the durability of our current fabrication method and prototypes and identify areas for future improvement, we conducted fatigue testing on the prototype with nine pouches (N9), made from TPU-coated nylon (Riverseal 70, 78Dtex, 70 g/m^2, Rivertex, Culemborg, The Netherlands). The prototype underwent cyclic testing in the MCL at ~60 BPM against mean afterload of 20 mmHg. Failure in this sample began after approximately 800 cycles (~13 minutes), evidenced by the appearance of air bubbles moving from the ventricle to the afterload chamber in the MCL. The failure point is indicated in Figures S10.a and S10.b. Following this, a slight reduction in maximum actuator pressure was observed (Figure S10.a), along with alterations in the afterload peak and minimum values, likely due to changes in flow dynamics and air leakage into the circuit.

The failure was caused by a small tear in the TPU layer near the sealing line (Figure S10.c), which allowed air to leak through nylon layer into the water stream. We consistently observed that among all the samples, tested during our study, a tear is occurring where the sealing line ends near the gaps for air channels as indicated in figure S10.c, suggesting a critical weak point. These findings indicate that, even with the current fabrication method, durability can be improved by eliminating gaps in the sealing lines and using individual air valves for each pouch to avoid stress concentrations at vulnerable points.

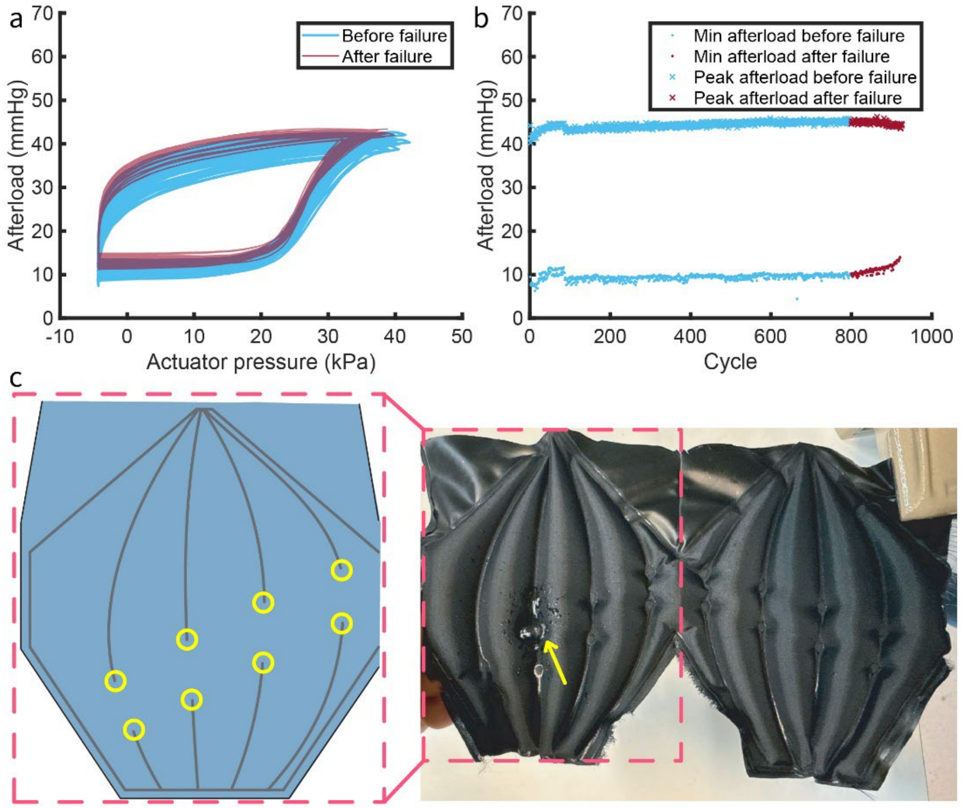

Figure S10. Results of fatigue experiments on N9 prototype. a) Change of actuator pressure and afterload after failure. b) Afterload peak and minimum values before and after failure. c) Points along the sealing line where the prototype is prone to tearing. We manually opened the ventricle after the fatigue experiment to observe the failure point on the inside of the ventricle.

Supplementary movies

Movie S1: Demonstration of a soft and efficient robotic fluidic transmission system that transfers low volume inputs at higher pressures into higher volume outputs at lower pressures.

Movie S2: Blocked-displacement experiment on pouch arrays with different number of pouches.

Movie S3: Step-by-step demonstration of the fast and cost-effective prototyping method.

Movie S4: Intraventricular deformation of the artificial ventricles with different number (size) of the pouch actuators.

Movie S5: In vitro assessment of a single LIMO Heart ventricle under physiological conditions and cyclic loading in a single-sided mock circulation loop

REFERENCES AND NOTES

1. M. Vaduganathan, G. A. Mensah, J. V. Turco, V. Fuster, G. A. Roth, The global burden of cardiovascular diseases and risk. *J. Am. Coll. Cardiol.* **80**, 2361–2371 (2022).

2. A. Vis, M. Arfaee, H. Khambati, M. S. Slaughter, J. F. Gummert, J. T. B. Overvelde, J. Kluin, The ongoing quest for the first total artificial heart as destination therapy. *Nat. Rev. Cardiol.* **19**, 813–828 (2022).

3. N. Melton, B. Soleimani, R. Dowling, Current role of the total artificial heart in the management of advanced heart failure. *Curr. Cardiol. Rep.* **21**, 142 (2019).

4. M. J. Slepian, Y. Alemu, J. S. Soares, R. G. Smith, S. Einav, D. Bluestein, The SynCardia™ total artificial heart: In vivo, in vitro, and computational modeling studies. *J. Biomech.* **46**, 266–275 (2013).

5. A. Carpentier, C. Latrémouille, B. Cholley, D. M. Smadja, J.-C. Roussel, E. Boissier, J.-N. Trochu, J.-P. Gueffet, M. Treillot, P. Bizouarn, D. Méléard, M.-F. Boughenou, O. Ponzio, M. Grimmé, A. Capel, P. Jansen, A. Hagège, M. Desnos, J.-N. Fabiani, D. Duveau, First clinical use of a bioprosthetic total artificial heart: Report of two cases. *Lancet* **386**, 1556–1563 (2015).

6. M. Arfaee, A. Vis, J. Kluin, Future technologies in total artificial heart development: Can a robot become as good as a donor heart? *Eur. Heart J.* **43**, 4970–4972 (2022).

7. M. Cianchetti, C. Laschi, A. Menciassi, P. Dario, Biomedical applications of soft robotics. *Nat. Rev. Mater.* **3**, 143–153 (2018).

8. P. Capsi-Morales, C. Piazza, M. G. Catalano, G. Grioli, L. Schiavon, E. Fiaschi, A. Bicchi, Comparison between rigid and soft poly-articulated prosthetic hands in non-expert myo-electric users shows advantages of soft robotics. *Sci. Rep.* **11**, 23952 (2021).

9. P. Polygerinos, Z. Wang, K. C. Galloway, R. J. Wood, C. J. Walsh, Soft robotic glove for combined assistance and at-home rehabilitation. *Rob. Auton. Syst.* **73**, 135–143 (2015).

10. H. K. Yap, J. H. Lim, F. Nasrallah, C.-H. Yeow, Design and preliminary feasibility study of a soft robotic glove for hand function assistance in stroke survivors. *Front. Neurosci.* **11**, 547 (2017).

11. L. Paternò, L. Lorenzon, Soft robotics in wearable and implantable medical applications: Translational challenges and future outlooks. *Front. Robot. AI* **10**, 10 (2023).

12. T. S. Hakky, R. Wang, G. D. Henry, The evolution of the inflatable penile prosthetic device and surgical innovations with anatomical considerations. *Curr. Urol. Rep.* **15**, 410 (2014).

13. A. Weymann, J. Foroughi, R. Vardanyan, P. P. Punjabi, B. Schmack, S. Aloko, G. M. Spinks, C. H. Wang, A. Arjomandi Rad, A. Ruhparwar, Artificial muscles and soft robotic devices for treatment of end-stage heart failure. *Adv. Mater.* **35** , 2207390 (2023).

14. L. G. Guex, L. S. Jones, A. X. Kohll, R. Walker, M. Meboldt, V. Falk, M. Schmid Daners, W. J. Stark, Increased longevity and pumping performance of an injection molded soft total artificial heart. *Soft Robot* **8**, 588–593 (2021).

15. N. H. Cohrs, A. Petrou, M. Loepfe, M. Yliruka, C. M. Schumacher, A. X. Kohll, C. T. Starck, M. Schmid Daners, M. Meboldt, V. Falk, W. J. Stark, A soft total artificial heart—First concept evaluation on a hybrid mock circulation. *Artif. Organs* **41**, 948–958 (2017).

16. E. T. Roche, M. A. Horvath, I. Wamala, A. Alazmani, S.-E. Song, W. Whyte, Z. Machaidze, C. J. Payne, J. C. Weaver, G. Fishbein, J. Kuebler, N. V. Vasilyev, D. J. Mooney, F. A. Pigula, C. J. Walsh, Soft robotic sleeve supports heart function. *Sci. Transl. Med.* **9**, eaaf3925 (2017).

17. E. T. Roche, R. Wohlfarth, J. T.B. Overvelde, N. V. Vasilyev, F. A. Pigula, D. J. Mooney, K. Bertoldi, C. J. Walsh, A bioinspired soft actuated material. *Adv. Mater.* **26**, 1200–1206 (2014).

18. M. A. Horvath, I. Wamala, E. Rytkin, E. Doyle, C. J. Payne, T. Thalhofer, I. Berra, A. Solovyeva, M. Saeed, S. Hendren, E. T. Roche, P. J. del Nido, C. J. Walsh, N. V. Vasilyev, An intracardiac soft robotic device for augmentation of blood ejection from the failing right ventricle. *Ann. Biomed. Eng.* **45**, 2222–2233 (2017).

19. C. J. Payne, I. Wamala, C. Abah, T. Thalhofer, M. Saeed, D. Bautista-Salinas, M. A. Horvath, N. V. Vasilyev, E. T. Roche, F. A. Pigula, C. J. Walsh, An implantable extracardiac soft robotic device for the failing heart: Mechanical coupling and synchronization. *Soft Robot.* **4**, 241–250 (2017).

20. C. J. Payne, I. Wamala, D. Bautista-Salinas, M. Saeed, D. Van Story, T. Thalhofer, M. A. Horvath, C. Abah, P. J. del Nido, C. J. Walsh, N. V. Vasilyev, Soft robotic ventricular assist device with septal bracing for therapy of heart failure. *Sci. Robot.* **2**, eaan6736 (2017).

21. A. X. Kohll, N. H. Cohrs, R. Walker, A. Petrou, M. Loepfe, M. Schmid Daners, V. Falk, M. Meboldt, W. J. Stark, Long-term performance of a pneumatically actuated soft pump manufactured by rubber compression molding. *Soft Robot* **6**, 206–213 (2019).

22. D. K. Molina, V. J. M. DiMaio, Normal organ weights in men: Part I—The heart. *Am. J. Forensic Med. Pathol.* **33**, 362–367 (2012).

23. A. G. Gheorghe, A. Fuchs, C. jacobsen, K. F. Kofoed, R. Møgelvang, N. Lynnerup, and J. Banner, Cardiac left ventricular myocardial tissue density, evaluated by computed tomography and autopsy. *BMC Med. Imaging* **19**, 29 (2019).

24. A. M. Maceira, S. K. Prasad, M. Khan, D. J. Pennell, Normalized left ventricular systolic and diastolic function by steady state free precession cardiovascular magnetic resonance. *J. Cardiovasc. Magn. Reson.* **8**, 417–426 (2006).

25. A. M. Maceira, S. K. Prasad, M. Khan, D. J. Pennell, Reference right ventricular systolic and diastolic function normalized to age, gender and body surface area from steady-state free precession cardiovascular magnetic resonance. *Eur. Heart J.* **27**, 2879–2888 (2006).

26. R. Niiyama, D. Rus, S. Kim, "Pouch motors: Printable/inflatable soft actuators for robotics," in *2014 IEEE International Conference on Robotics and Automation (ICRA)* (IEEE, 2014).

27. M. Arfaee, J. Kluin, J. T. B. Overvelde, "Modeling the behavior of elastic pouch motors," in *2023 IEEE International Conference on Soft Robotics (RoboSoft)* (IEEE, 2023).

28. K. Narumi, H. Sato, K. Nakahara, Y. Seong, K. Morinaga, Y. Kakehi, R. Niiyama, Y. Kawahara, Liquid pouch motors: Printable planar actuators driven by liquid-to-gas phase change for shape-changing interfaces. *IEEE Robot. Autom. Lett.* **5**, 3915–3922 (2020).

29. X. Wang, S. K. Mitchell, E. H. Rumley, P. Rothemund, C. Keplinger, High-strain Peano-HASEL actuators, *Adv. Funct. Mater.* **30**,1908821 (2020).

30. D. Sidebotham, I. J. Le Grice, "Chapter 1 - Physiology and pathophysiology," in *Cardiothoracic Critical Care*, D. Sidebotham, A. McKee, M. Gillham, J. H. Levy, Eds. (Butterworth-Heinemann, 2007), pp 3–27.

31. M. Ragosta, J. L. W. Kennedy, "Chapter 2 - Normal waveforms, artifacts, and pitfalls," in *Textbook of Clinical Hemodynamics*, M. Ragosta, Ed. (Elsevier, ed. 2, 2018), pp. 17–55.

32. G. Liljestrand, E. Lysholm, G. Nylin, C. G. Zachrisson, The normal heart volume in man. *Am. Heart J.* **17**, 406–415 (1939).

33. D. Bamira, M. H. Picard, "Imaging: Echocardiology—Assessment of cardiac structure and function," in *Encyclopedia of Cardiovascular Research and Medicine*, R. S. Vasan, D. B. Sawyer, Eds. (Elsevier, 2018), pp. 35–54.

34. Institute of Medicine Committee on Social Security Cardiovascular Disability Criteria, *Cardiovascular Disability: Updating the Social Security Listings* (National Academies Press, 2010).

35. A. Corsini, L. Cercenelli, M. Zecchi, E. Marcelli, I. Corazza, "Chapter 30 - Basic hemodynamic parameters," in *Advances in Cardiovascular Technology*, J. H. Karimov, K. Fukamachi, M. Gillinov, Eds. (Academic Press, 2022), pp. 463–474.

36. M. T. L. Edmund, G. Laurent, S. Olivier, M. David, S. Laurent, J. Xavier, L. Frederic, G. Sven, S. C. David, S. Gérald, H. Marc, C. Denis, H. Philippe, Resting pulmonary artery pressure of 21–24 mmHg predicts abnormal exercise haemodynamics. *Eur. Respir. J.* **47**, 1436–1444 (2016).

37. S. Yousefi, H. Borna, A. Rohani Shirvan, C. Wen, A. Nouri, Surface modification of mechanical heart valves: A review. *Eur. Polym. J.* **205**, 112726 (2024).

38. S. Loerakker, G. Argento, C. W. J. Oomens, F. P. T. Baaijens, Effects of valve geometry and tissue anisotropy on the radial stretch and coaptation area of tissue-engineered heart valves. *J. Biomech.* **46**, 1792–1800 (2013).
